# Successful management of ischaemic symptoms in a patient with asymmetric septal hypertrophy: a grand round case report

**DOI:** 10.1093/ehjcr/ytae213

**Published:** 2024-04-25

**Authors:** Damirbek Osmonov, Azimbek Toktosunov, Aida Toktogulova, Dilrabo Kasymova, Unal Mustafa

**Affiliations:** Department of Cardiology, Bicard Clinic, Tynystanov st 2/1, Bishkek 72000, Kyrgyzstan; Department of Cardiology, Bicard Clinic, Tynystanov st 2/1, Bishkek 72000, Kyrgyzstan; Department of Cardiology, Bicard Clinic, Tynystanov st 2/1, Bishkek 72000, Kyrgyzstan; Department of Anesthesiology, Bicard Clinic, Tynystanov st 2/1, Bishkek 72000, Kyrgyzstan; Department of Cardiac Surgery, Bicard Clinic, Tynystanov st 2/1, Bishkek 72000, Kyrgyzstan

**Keywords:** Hypertrophic cardiomyopathy, Asymmetric septal hypertrophy, Ethyl alcohol ablation, Myocardial bridging, Left ventricular outflow tract obstruction, Case report

## Abstract

**Background:**

Hypertrophic cardiomyopathy (HCM) is a genetic heart disease that can lead to heart failure, atrial fibrillation, and ischaemic symptoms. Managing patients with HCM and ischaemic symptoms is challenging, and several treatment options have been proposed.

**Case summary:**

A 30-year-old male patient presented with severe chest pain that had been ongoing for more than 30 min at rest. He was diagnosed with HCM and had periodic chest pain since the age of 14. He underwent two separate ethyl alcohol ablations of the first septal branches of the left anterior descending and posterior descending arteries, which relieved his symptoms.

**Discussion:**

This case report highlights the challenges in managing patients with HCM and ischaemic symptoms. In this patient, the use of ethyl alcohol ablation was effective in reducing left ventricular outflow tract obstruction and improving symptoms. Ethyl alcohol ablation is a minimally invasive procedure that has been shown to be effective in symptomatic patients with HCM. Overall, this case report emphasizes the importance of individualized treatment for patients with HCM and the potential benefits of alcohol ablation in this population.

**Learning points**

- Management of ischaemic symptoms in patients with hypertrophic cardiomyopathy (HCM) can be challenging, and individualized treatment options may be necessary.
- Ethyl alcohol ablation is a minimally invasive procedure that has been shown to be effective in reducing left ventricular outflow tract obstruction and improving ischaemic symptoms in patients with HCM.
- In some cases, additional interventions may be necessary to manage symptoms in patients with HCM, such as myotomy for relief of a muscular bridge. Close follow-up and careful monitoring are important to ensure optimal management of these patients.

## Primary specialties involved other than cardiology

Cardiac surgery, anesthesiology, and electrophysiology.

## Introduction

Hypertrophic cardiomyopathy (HCM) is a genetic heart disease characterized by hypertrophy and abnormal arrangement of myocardial fibres. It is the most common cause of sudden cardiac death (SCD) in young athletes and can lead to heart failure, atrial fibrillation, and ischaemic symptoms.^1^ Asymmetric septal hypertrophy (ASH), a subtype of HCM, is characterized by asymmetric thickening of the septum, which can result in left ventricular outflow tract obstruction (LVOTO) and mitral regurgitation.^2^ In this case report, we describe the successful management of ischaemic symptoms in a patient with ASH using ethyl alcohol ablation of the septal branches of the left anterior descending and posterior descending arteries.

## Timeline

**Table:** 

Time point	Event
2002 (first diagnosis)	Patient underwent an echocardiographic evaluation because of systolic heart murmur. Hypertrophic cardiomyopathy with left ventricular outflow tract obstruction (maximal gradient 40 mmHg) was detected.
2005 (symptom onset)	Patient started to feel chest pain.
2009 (coronary angiography)	Because of long-standing chest pain, coronary angiography was done. Myocardial bridging with 50% stenosis during systole was revealed at mid-portion of left anterior descending artery.
2014–21 (multiple hospitalizations)	The patient was hospitalized multiple times in cardiology clinics of several countries like Russia, Turkey and Kyrgyzstan. They had been treated conservatively without any interventions.
April 2021 (first episode of atrial fibrillation and syncope)	The patient presented to the emergency room with syncope and chest pain that had been ongoing for more than 30 min at rest. Atrial fibrillation with a high ventricular rate was detected on ECG. The pulse was 128 b.p.m., and blood pressure was 80/50 mmHg. Emergency DC cardioversion was performed with 150 J.
April 2021 (workup)	Troponin was slightly elevated [0.3 ng/mL (normal range: <0.2 ng/mL)]. An echocardiography showed a thickened left ventricular septum up to 38 mm, and left ventricular outflow tract gradient was 40 mmHg, posterior wall thickness was 10 mm with an ejection fraction of 80%.
April 2021 (management with ethyl alcohol ablation)	Coronary angiography revealed very well-developed septal branches, completely collapsing during systole, of the left anterior descending (LAD) and posterior descending (PDA) branch of the right coronary artery (RCA). Ethyl alcohol ablation of the first septal branch of LAD was performed under general anaesthesia with 2 mL of 95% ethyl alcohol.
April 2021 (control workup after the alcohol ablation)	Troponin was elevated up to 15 ng/mL (normal range: <0.2 ng/mL). An echocardiography showed decreased thickness of LV septum to 28 mm and maximal LVOT gradient to 16 mmHg.
April 2021 (discharge from the hospital)	The patient was free of chest pain and was discharged uneventfully, 4 days after the alcohol ablation, on acetyl salicylic acid 75 mg, bisoprolol 5 mg, amiodarone 200 mg, and clopidogrel 75 mg.
July and September 2021 (hospitalizations)	The patient was free of chest pain for three months after the procedure. Then he started to feel moderate to severe chest pain at rest. He was hospitalized twice with chest pain and elevated troponin up to 0.45 ng/mL.
November 2021 (control coronary angiography)	Coronary angiography revealed a negative correlation between pulse and coronary blood flow rate in the left anterior descending artery (LAD), as measured by transvenous pacemaker. Also, stenosis of the muscular bridge in mid LAD increased up to 90% during systole. At his own pulse of 50 b.p.m., coronary flow was normal. At pulses 70, 100, and 120 b.p.m., the coronary flow was TIMI II, TIMI I, and TIMI 0, respectively.
November 2021 (myotomy)	Muscular bridge relieving operation was performed. Unfortunately, it did not relieve the chest pain significantly.
December 2021 (second ethyl alcohol ablation)	Ethyl alcohol ablation from the right coronary artery posterior descending arteries first septal branch with 2.5 mL of 95% alcohol.
December 2021 (control investigation and discharge)	ECG detected complete right bundle branch block. Echocardiography showed a thickness of LV septum 21 mm and maximal LVOT gradient 7 mmHg. The patient was free of chest pain and discharged uneventfully.
January, March, and June 2022 (follow-up)	The patient had no complaints and was active. Follow-up ECG at the first, third, and sixth months after discharge showed a complete right bundle branch block and first-degree atrioventricular block without any further progression of atrioventricular block.

## Patient presentation

A 30-year-old male patient arrived at the emergency room with persistent chest pain lasting over 30 min while at rest. His pulse registered at 55 b.p.m., and his blood pressure measured 100/60 mmHg. An electrocardiogram (ECG) exhibited a sinus rhythm with indications of left ventricular hypertrophy. Results from renal and hepatic function tests appeared normal, but troponin levels were slightly elevated at 0.43 ng/mL (normal range: <0.2 ng/mL). An echocardiography confirmed the presence of asymmetric septal hypertrophic cardiomyopathy.

**Figure 1:**
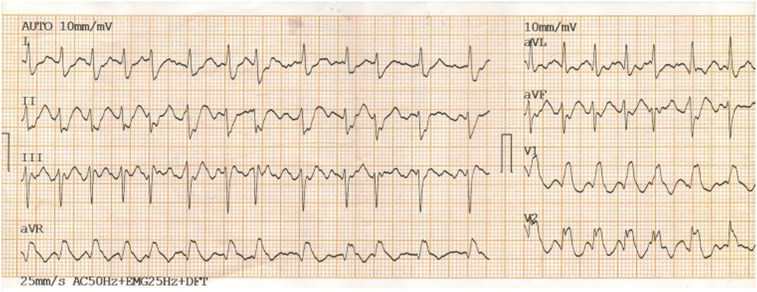
Following the episode of syncope, an electrocardiogram (ECG) was performed and indicated the presence of atrial fibrillation with a rapid ventricular response.

**Figure 2:**
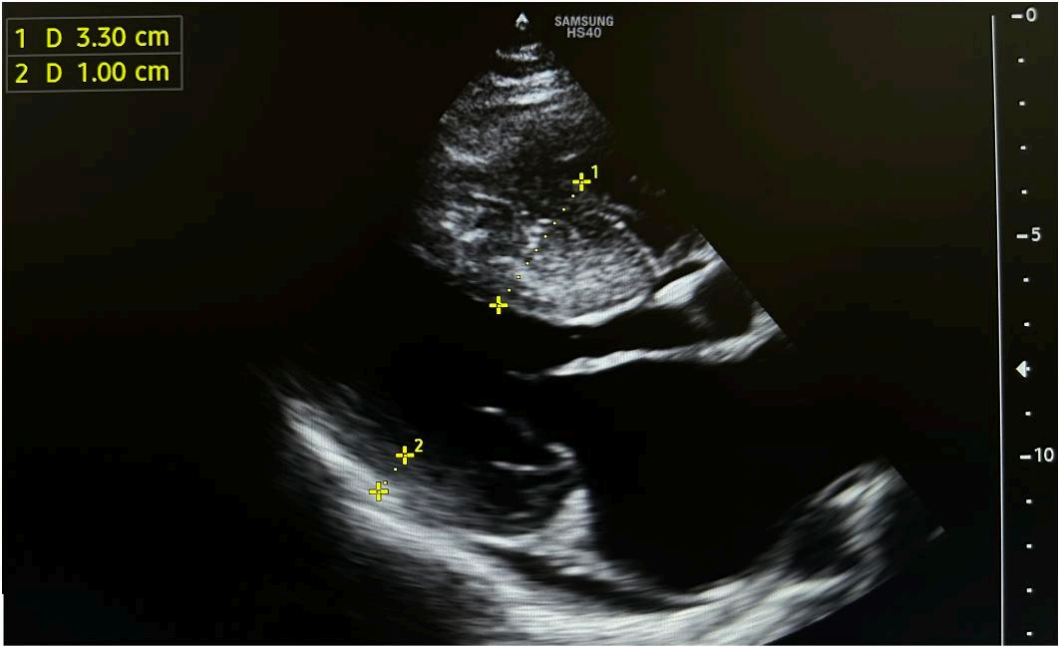
The initial echocardiography conducted prior to the ethanol ablation procedure revealed a thickened left ventricular septum, measuring up to 33 mm, causing obstruction within the left ventricular outflow tract up to 40 mmHg of gradient.

**Figure 3:**
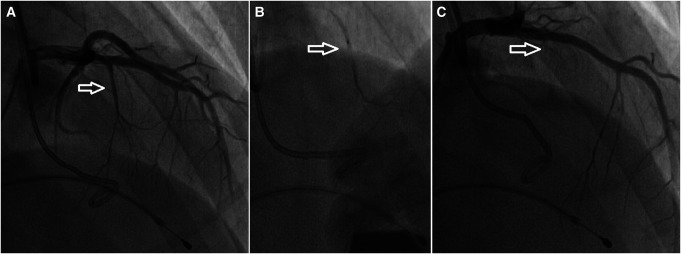
Alcohol septal ablation of the first septal branch of left anterior descending artery is shown: the dominant first septal branch (arrow) (A); an over-the-wire balloon (arrow) is inflated in the dominant first septal branch (B); occlusion of septal branch (arrow) after the alcohol ablation (C).

**Figure 4:**
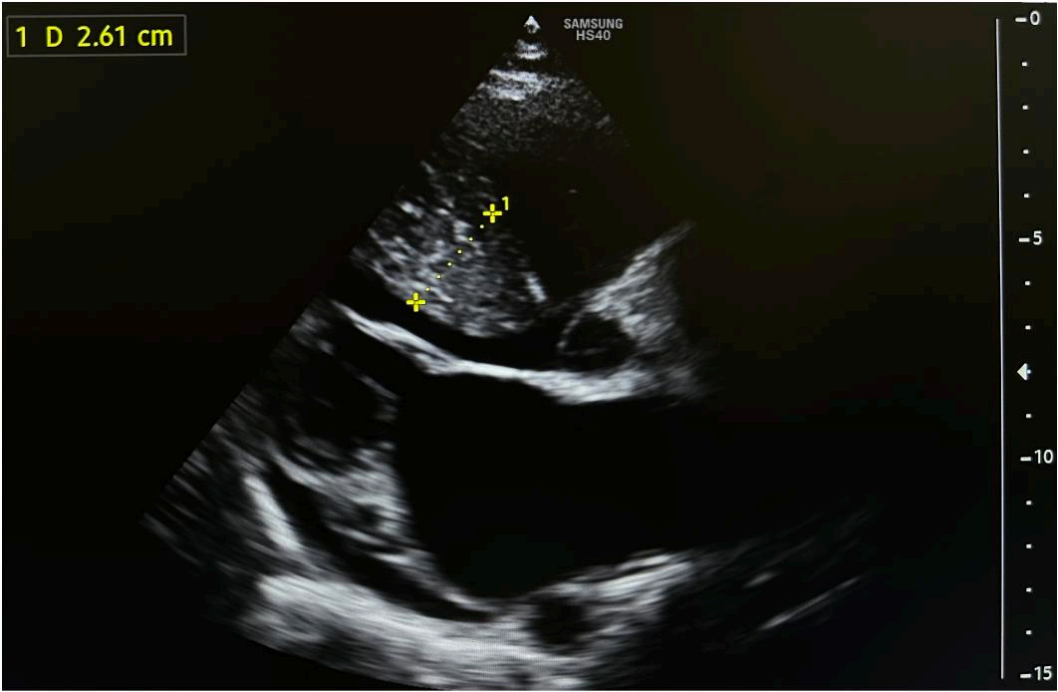
Subsequent to the first ethanol ablation, a follow-up echocardiography demonstrated a reduction in the thickness of the left ventricular (LV) septum to 26 mm, alongside a decrease in the left ventricular outflow tract (LVOT) gradient to 16 mmHg.

A diagnosis of HCM with LVOTO was established when he was 11 years old. He began experiencing periodic refractory chest pain at the age of 14. Over the years, he was admitted to cardiology departments and received medical treatment in multiple countries. In April 2021, the patient suffered syncope. An ECG (*Figure 1*) revealed atrial fibrillation with a high ventricular rate. In response, emergency DC cardioversion was conducted using 150 J due to a pulse of 128 b.p.m. and a blood pressure of 80/50 mmHg. Despite achieving sinus rhythm with a heart rate of 60–70 b.p.m., the patient continued to endure prolonged bouts of resting chest pain, which only responded to opioids. Subsequent echocardiography indicated a thickened LV septum measuring up to 33 mm, mild mitral regurgitation with an LVOT gradient of 40 mmHg (*Figure 2*). These measurements were taken at rest. Due to the patient’s severe chest pain even at rest, exercise testing was not performed. A coronary angiography showed well-developed septal branches that collapsed entirely during systole within the left anterior descending (LAD) and posterior descending (PDA) branches of the right coronary artery (RCA). Additionally, a muscular bridge was observed on mid LAD, contributing to 50% stenosis during systole. To address this, an ethyl alcohol ablation of the first septal branch of the LAD was performed in April 2021 under general anaesthesia, utilizing 2 mL of 95% ethyl alcohol (*Figure 3*). Post-procedure assessment confirmed complete occlusion of the septal branch (*Figure 3C*), resulting in significant relief from angina. Follow-up echocardiography indicated a decrease in LV septum thickness to 26 mm and an LVOT gradient reduction to 16 mmHg at rest (*Figure 4*). Upon discharge, the patient’s medication regimen included acetylsalicylic acid 75 mg, bisoprolol 5 mg, amiodarone 200 mg, and clopidogrel 75 mg.

The patient remained free of chest pain for three months after the first alcohol ablation procedure. However, he then began to experience moderate to severe chest pain at rest. His pulse and blood pressure at the moment of chest pain were 70 b.p.m. and 90–100/70 mmHg, respectively. He was hospitalized twice with elevated troponin levels, up to 0.45 ng/mL. In November 2021, a coronary angiography was conducted, revealing a negative correlation between pulse and coronary blood flow rate in the left anterior descending artery (LAD), as measured by a transvenous pacemaker. Furthermore, stenosis of the muscular bridge in mid LAD had increased up to 90%. At his own pulse of 50 b.p.m., coronary flow was normal with complete occlusion of septal branches during systole (see Supplementary material online, *Video S1*). At pulse rates of 70, 100, and 120 b.p.m., the coronary flow was categorized as TIMI II, TIMI I, and TIMI 0, respectively (see Supplementary material online, *Videos S2*, *S3*, and *S4*, respectively). A decision was made to relieve the muscular bridge through myotomy in November 2021, but this procedure did not significantly alleviate the chest pain.

**Figure 5:**
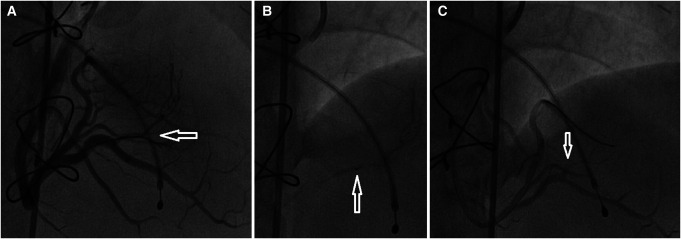
Alcohol septal ablation of the first septal branch of posterior descending artery is shown: the dominant first septal branch (arrow) (*A*); an over-the-wire balloon (arrow) is inflated in the dominant first septal branch (B); occlusion of septal branch (arrow) after the alcohol ablation (C).

**Figure 6:**
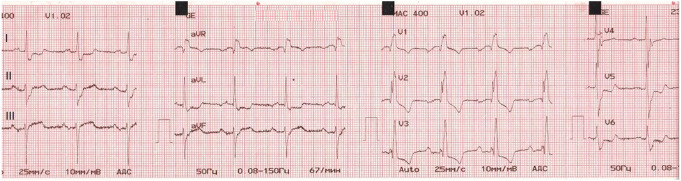
Following the completion of the two ethanol ablation procedures, an electrocardiogram (ECG) showed a complete right bundle branch block accompanied by a first-degree atrioventricular block.

**Figure 7:**
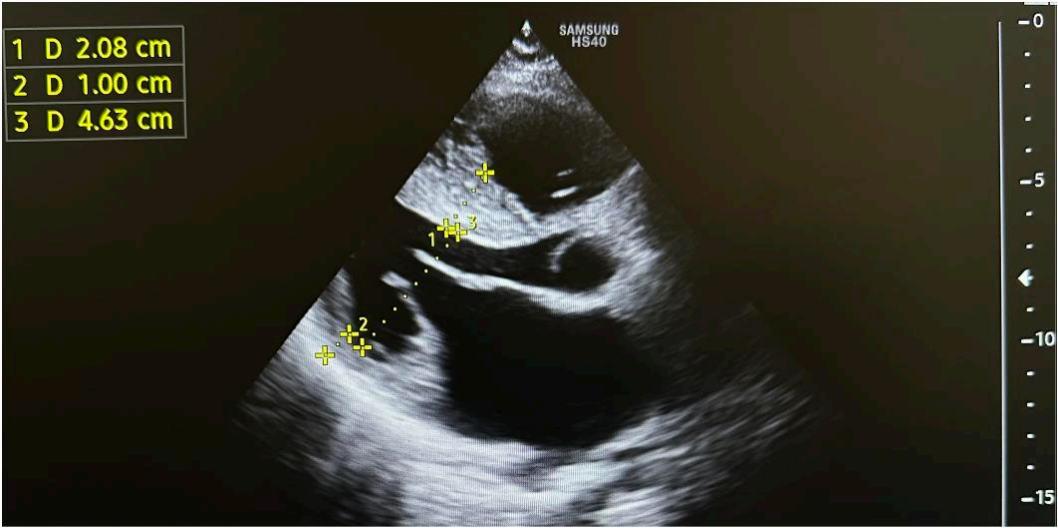
After the completion of the two ethanol ablation procedures, a subsequent echocardiography revealed an interventricular wall thickness of 21 mm and a gradient of 7 mmHg at the LVOT.

In December 2021, during the last hospitalization, we decided to perform the second alcohol ablation using 2.5 mL of 95% ethanol on the first septal branch of the right coronary artery’s posterior descending artery (*Figure 5*). Following the procedure, the chest pain disappeared. A subsequent ECG detected a complete right bundle branch block with the first-degree atrioventricular block (*Figure 6*), and an echocardiography revealed an interventricular wall thickness of 21 mm and a 7 mmHg gradient at the LVOT at rest (*Figure 7*). Follow-up ECGs at the first, third, and sixth months after discharge consistently showed a complete right bundle branch block without progression of atrioventricular block. During this period, he remained free of chest pain. According to the HCM Risk-SCD risk score, the patient had a 4.14% risk of SCD at 5 years, indicating an intermediate risk with a Class IIb indication for implantable cardioverter defibrillator (ICD) implantation.^3^ We did not implant him an ICD. His current medications include bisoprolol 5 mg once daily, acetylsalicylic acid 75 mg once daily, nicorandil 10 mg once daily, and disopyramide 150 mg twice daily.

## Discussion

Hypertrophic cardiomyopathy is a genetically inherited heart condition commonly encountered in clinical practice. It is usually asymptomatic, but some patients may experience chest pain, dyspnoea, syncope, or SCD.^1^ Our patient presented with recurrent chest pain and was diagnosed with ASH on echocardiography.^4^

The management of patients with ASH and ischaemic symptoms is challenging, and several treatment options have been proposed, including medical therapy, septal reduction therapy, and dual-chamber pacing.^5^ Our patient was initially treated with DC cardioversion and medical therapy, but he continued to have long-lasting chest pain attacks despite sinus rhythm and well-controlled blood pressure. ESC Guidelines on diagnosis and management of hypertrophic cardiomyopathy recommend an invasive treatment to reduce LVOTO in patients with an LVOT gradient ≥ 50 mmHg, moderate to severe symptoms [New York Heart Association (NYHA) functional Class III–IV] and/or recurrent exertional syncope in spite of maximally tolerated drug therapy.^3^ Despite the gradient never reaching ≥ 50 mmHg, we opted for this procedure due to the patient’s significant and refractory symptoms, which posed a substantial impairment to his quality of life. Although the gradient never reached >50 mmHg at rest, we suspected that exercise could potentially induce a higher gradient. However, due to the severity of the patient’s symptoms, exercise testing was not feasible.

Ethyl alcohol ablation leads to localized myocardial necrosis and thinning of the hypertrophied septum. It has been shown to be effective in reducing LVOTO and improving symptoms in patients with HCM and refractory heart failure or ischaemic symptoms.^5^

While the 2023 ESC Guidelines for the management of cardiomyopathies may caution against alcohol septal ablation in patients with septal thickness close to 30 mm,^6^ the decision in this case was likely influenced by the patient’s refractory symptoms and the lack of response to conservative management. In our case, the first ethyl alcohol ablation reduced LVOTO and relieved the patient’s angina for three months, but the symptoms recurred thereafter. The second ethyl alcohol ablation of the first septal branch of the RCA PDA was successful in improving the patient’s symptoms and echocardiographic parameters. However, the procedure carries a risk of complete heart block, ventricular arrhythmias, and coronary artery dissection/thrombosis. Therefore, it should be performed in experienced centres with careful patient selection and monitoring.^7^ A complete right bundle branch block with first-degree atrioventricular block occurred in the case we presented after the second alcohol ablation.

Surgical septal myectomy has been shown to improve symptoms and survival in patients with obstructive HCM.^8^ Several meta-analyses have shown that both surgery and septal alcohol ablation improve functional status with a similar procedural mortality.^9,10^ However, it should be noted that while surgical myectomy is generally considered safe and effective, it is not appropriate for all patients with ASH and a muscular bridge. The decision to undergo surgery should be made on a case-by-case basis after careful consideration of the patient’s overall health and individual needs.

Myocardial bridging has been associated with the severity of cardiac disease and an increased risk of SCD in paediatric patients with HCM.^11,12^ Also, arrhythmia and myocardial infarction can be seen; these can cause sudden death, especially in athletes and young people. However, the largest reported population of adult patients with HCM who have myocardial bridging observed no increased risk of death, including SCD, among patients with HCM who had myocardial bridging, as compared with HCM patients without bridging.^13^ In our case, there was no improvement of ischaemic symptoms after the operation for relieving a myocardial bridging.

In this case, although the patient had an intermediate risk of SCD based on the HCM Risk-SCD risk score, a decision was made not to implant an ICD.^3^ This decision was influenced by factors such as the patient’s arrhythmic profile, featuring paroxysmal atrial fibrillation, and the absence of a significant family history of SCD. Additionally, the patient experienced significant symptomatic relief post-ethyl alcohol ablation procedures, potentially reducing the immediate need for an ICD.

This case report emphasizes the importance of individualized treatment and the role of ethyl alcohol ablation as an effective, minimally invasive intervention for symptomatic patients with ASH. However, it also highlights the complexity of managing patients with multiple cardiac issues, such as AF and myocardial bridging, which may contribute to recurrent symptoms even after successful interventions. Given that the AF was of recent onset and reverted to sinus rhythm with the first attempt of moderate energy direct current cardioversion (DCCV), and considering the patient’s low risk of stroke as assessed by the CHA2DS2-VASc score, we opted for a ‘wait-and-see’ approach until the second episode of AF before considering initiation of anticoagulation therapy. Clinicians need to consider the interplay of these conditions and adopt a holistic approach to optimize patient outcomes.

Furthermore, this report underscores the need for ongoing research and long-term follow-up to better understand the outcomes and effects of interventions in patients with ASH and associated comorbidities. A collaborative effort between cardiologists, electrophysiologists, cardiac surgeons, and genetic counsellors is essential to develop comprehensive treatment strategies tailored to individual patient profiles. By addressing these challenges and implementing evidence-based interventions, clinicians can enhance the management and outcomes of patients with ASH and ischaemic symptoms.

## Supplementary Material

ytae213_Supplementary_Data

## Data Availability

The data used to support the findings of this study are available from the corresponding author upon request.

## Lead author biography

I graduated from the Hacettepe University, Faculty of Medicine, Ankara, Turkey, 1999–2005. I completed my cardiology residency from the Siyami Ersek Thoracic and Cardiovascular Surgery Training and Education Center, Istanbul, Turkey: April 2006–April 2012. I am involved in interventional cardiology and arrhythmology.
